# Prognostic value of PD-L1 expression in recurrent renal cell carcinoma after nephrectomy: a secondary analysis of the ARCHERY study

**DOI:** 10.1007/s10147-022-02256-z

**Published:** 2022-12-19

**Authors:** Satoshi Tamada, Masahiro Nozawa, Kojiro Ohba, Ryuichi Mizuno, Atsushi Takamoto, Chisato Ohe, Takuya Yoshimoto, Yuki Nakagawa, Tamaki Fukuyama, Nobuaki Matsubara, Go Kimura, Yoshihiko Tomita, Norio Nonomura, Masatoshi Eto

**Affiliations:** 1 Department of Urology, Bell Land General Hospital, Higashiyama 500-3, Naka-Ku, Sakai City, Osaka 599-8247 Japan; 2 Department of Urology, Faculty of Medicine, Kindai University, 377-2 Ohno-Higashi, Osaka-Sayama, Osaka 589-8511 Japan; 3 Department of Urology and Renal Transplantation, Nagasaki University Hospital, 1-7-1 Sakamoto, Nagasaki, 852-8501 Japan; 4 Department of Urology, Keio University School of Medicine, 35 Shinanomachi, Shinjuku-Ku, Tokyo, 160-8582 Japan; 5 Department of Urology, Fukuyama City Hospital, 5-23-1 Zao-Cho, Fukuyama, Hiroshima 721-8511 Japan; 6 Department of Pathology, Kansai Medical University, 2-3-1 Shinmachi, Hirakata, Osaka 573-1191 Japan; 7 Clinical Development Division, Chugai Pharmaceutical Co, Ltd, 1-1 Nihonbashi-Muromachi, 2-Chome Chuo-Ku, Tokyo, 103-8324 Japan; 8 Department of Medical Oncology, National Cancer Center Hospital East, 6-5-1 Kashiwanoha, Kashiwa-Shi, Chiba, 277-8577 Japan; 9 Department of Urology, Nippon Medical School, 1-1-5 Sendagi, Bunkyo-Ku, Tokyo, 113-8603 Japan; 10 Departments of Urology and Molecular Oncology, Niigata University Graduate School of Medical and Dental Sciences, 1-757 Asahimachi-Dori, Chuo-Ku, Niigata, 951-8510 Japan; 11 Department of Urology, Osaka University Graduate School of Medicine, 2-2 Yamadaoka, Suita, Osaka 565-0871 Japan; 12 Department of Urology, Graduate School of Medical Sciences, Kyushu University, 3-1-1 Maidashi, Higashi-Ku, Fukuoka, 812-8582 Japan

**Keywords:** ARCHERY, Nephrectomy, Nuclear grade, Prognosis, Programmed death-ligand 1, Renal cell carcinoma

## Abstract

**Background:**

Nephrectomy is a curative treatment for localized renal cell carcinoma (RCC), but patients with poor prognostic features may experience relapse. Understanding the prognostic impact of programmed death-ligand 1 (PD-L1) expression in patients who underwent nephrectomy for RCC may aid in future development of adjuvant therapy.

**Methods:**

Of 770 surgical specimens collected from Japanese patients enrolled in the ARCHERY study, only samples obtained from patients with recurrent RCC after nephrectomy were examined for this secondary analysis. Patients were categorized into low- and high-risk groups based on clinical stage and Fuhrman grade. Time to recurrence (TTR) and overall survival (OS) were analyzed.

**Results:**

Both TTR and OS were shorter in patients with PD-L1-positive than -negative tumors (median TTR 12.1 vs. 21.9 months [HR 1.46, 95% CI 1.17, 1.81]; median OS, 75.8 vs. 97.7 months [HR 1.32, 95% CI 1.00, 1.75]). TTR and OS were shorter in high-risk patients with PD-L1-positive than -negative tumors (median TTR 7.6 vs. 15.3 months [HR 1.49, 95% CI 1.11, 2.00]; median OS, 55.2 vs. 83.5 months [HR 1.53, 95% CI 1.06, 2.21]) but not in low-risk patients.

**Conclusions:**

This ARCHERY secondary analysis suggests that PD-L1 expression may play a role in predicting OS and risk of recurrence in high-risk patients with localized RCC.

Clinical Trial Registration: UMIN000034131.

**Supplementary Information:**

The online version contains supplementary material available at 10.1007/s10147-022-02256-z.

## Introduction

In 2019, 32,200 patients in Japan were projected to have cancer of the kidney or another urinary organ combined, with an age-standardized rate of 13.1 for males and 4.7 for females per 100,000 people [1]. The latest available Japanese cancer statistics, obtained between 2009 and 2011, indicate that 56% of patients with cancer of the kidney and other urinary organs (except the bladder) had localized disease at diagnosis [2].

Although nephrectomy is a curative treatment for renal cell carcinoma (RCC), patients may experience relapse, especially if they are at high risk (i.e., poor prognostic factors, including high nuclear/Fuhrman grade and high tumor [T] stage) [3]. The University of California, Los Angeles Integrated Staging System (UISS) model classifies patients by risk levels based on T stage, node (N) stage, Fuhrman grade, and Eastern Cooperative Oncology Group performance status [4]. Per the UISS, the 5-year recurrence-free rates in 559 patients who underwent surgery for localized and locally advanced RCC were 90.4, 61.8, and 41.9% in the low-, intermediate-, and high-risk groups, respectively [4]. Therefore, adjuvant treatment is necessary, especially for patients at high risk.

Many phase 3 randomized controlled trials (RCTs) have examined the effect of immunotherapy, including programmed death-1 (PD-1) and programmed death-ligand 1 (PD-L1) blockade in the adjuvant setting for localized high-risk RCC [5, 6]. In the KEYNOTE-564 study, postoperative treatment with pembrolizumab significantly improved the disease-free survival of patients with localized clear cell RCC versus placebo (HR 0.68; 95% CI 0.53, 0.87; one-sided *P* = 0.0010). Patients enrolled in KEYNOTE-564 were intermediate-high risk, high risk, or M1 with no evidence of disease ≤ 1 year from nephrectomy. This result suggests that PD-(L)1 blockade is a promising adjuvant therapy for RCC [7]. Therefore, elucidating the prognostic value of PD-L1 in patients with localized RCC will be useful in developing future adjuvant treatment strategies (e.g., patient selection).

The prognostic impact of PD-L1 expression on tumor-infiltrating immune cells (IC) in patients with recurrent or metastatic RCC treated with systemic therapy was previously investigated in a retrospective study, ARCHERY [8]. Median OS for PD-L1-positive patients and PD-L1-negative patients was 30.9 months (95% CI 25.5, 35.7) and 37.5 months (95% CI 34.0, 42.6), respectively (HR 1.04; 90% CI 0.89, 1.22; *P* = 0.65]; stratified by Memorial Sloan Kettering Cancer Center [MSKCC] risk and liver metastases). Results showed that PD-L1 expression was not an independent prognostic marker after first-line (1L) treatment because it was associated with other factors, especially MSKCC risk status.

This secondary analysis of ARCHERY investigated the prognostic impact of PD-L1 IC status in patients who underwent nephrectomy for localized RCC. We evaluated time to recurrence (TTR) and OS after nephrectomy. The prognostic impact in high-risk patients was also explored because several ongoing phase 3 studies are investigating the use of checkpoint inhibitors (CPIs) in the adjuvant setting in high-risk patients with RCC who underwent nephrectomy [6]. In addition, the prognostic effect of nuclear grade alone and in combination with PD-L1 status was examined. This secondary analysis will provide new insights into the role of PD-L1 expression in prognosis and risk of recurrence in localized RCC, potentially aiding future adjuvant treatment strategies.

## Patients and methods

### Patients

In the ARCHERY study, patients with metastatic or recurrent RCC who started 1L treatment between January 2010 and December 2015 were enrolled [8]. Patients underwent either cytoreductive nephrectomy or radical nephrectomy for localized disease. This exploratory secondary analysis comprised patients enrolled in ARCHERY who had stored formalin-fixed paraffin-embedded surgical samples of the primary lesion. Between November 2018 and June 2019, specimens were obtained from 29 Japanese study sites. Exclusion criteria included coexisting post-nephrectomy malignancies or first-line treatment with anti-cytotoxic T-lymphocyte-associated antigen 4/anti-PD-(L)1 for recurrent/metastatic RCC. All patients selected for this analysis were recurrent cases.

This study is registered in the UMIN Clinical Trials Registry (UMIN000034131) and was performed after approval by each institutional review board of the 29 study sites. Furthermore, approval was obtained from the institutional review board of MINS, a non-profit organization. Informed consent was obtained from all the participants, and this study was conducted in accordance with the Declaration of Helsinki.

### Study design

ARCHERY is a multicenter, retrospective study that studied the prognostic effect of PD-L1 status on overall survival (OS) in patients with recurrent or metastatic RCC who had received systemic therapy. In this secondary analysis, the prognostic value of PD-L1 expression was examined in patients with recurrent RCC after nephrectomy.

The objectives included time to recurrence (TTR; defined as the time from nephrectomy to the date of recurrence) and OS (defined as the time from nephrectomy to death from any cause) by PD-L1 status.

### Subgroup analyses

Exploratory subgroup analyses included TTR and OS by PD-L1 status and risk level. Patients were categorized into high (stage III/IV or stage II and Fuhrman grade 4 at initial diagnosis) and low risk (stage I or stage II and Fuhrman grade ≤ 3 at initial diagnosis). Clinical stage was used instead of T stage because information on T stage was not collected in ARCHERY since the study focused on OS after 1L treatment of recurrent or metastatic RCC. The eligibility for high risk was based on current ongoing phase 3 trials evaluating CPIs in high-risk localized RCC [6]. ARCHERY showed a correlation between PD-L1 expression and nuclear grade [8]. Of note, nuclear/Fuhrman grade was a risk factor in the four commonly used localized RCC prognostic models, including the MSKCC postoperative nomogram [3]. Hence, subgroup analyses of TTR and OS by PD-L1 status and nuclear grade (both Fuhrman grade and World Health Organization/International Society of Urological Pathology [WHO/ISUP] grade) was conducted in the secondary analysis population and each risk group.

### Pathology and immunohistochemistry

Representative formalin-fixed paraffin-embedded samples were selected by pathologists in each institution and evaluated by a central pathologist [8]. PD-L1 expression was evaluated by immunohistochemistry using the SP142 assay (VENTANA Medical Systems, Inc, Tucson, Arizona, USA) [9]. Based on PD-L1 expression levels on ICs, patients were classified as either PD-L1 negative (IC0, IC < 1%) or positive (further divided into IC1 [IC ≥ 1% but < 5%], IC2 [IC ≥ 5% but < 10%], or IC3 [IC ≥ 10%]). Immune phenotype assessment (excluded, inflamed, and desert) was performed using CD8 immunostaining [10].

### Statistical analyses

TTR and OS were estimated using Kaplan–Meier analysis, the CI of median (m) TTR and OS were estimated using the Brookmeyer–Crowley technique [11] after complementary log–log transformation, and the hazard ratio (HR) was estimated using the Cox proportional hazards model.

## Results

### Patient characteristics

Of the 770 patients enrolled in ARCHERY, 381 who underwent nephrectomy for localized RCC were analyzed in this secondary analysis (Table 1) [8].

**Table 1:** Baseline clinicopathological characteristics at the time of diagnosis or nephrectomy in patients who underwent nephrectomy by PD-L1 status

Characteristics, *n* (%)	PD-L1 positive^a^ (*n* = 120)	PD-L1 negative^b^ (*n* = 261)	Total (*N* = 381)
Male	88 (73.3)	201 (77.0)	289 (75.9)
Female	32 (26.7)	60 (23.0)	92 (24.1)
*Age at the time of nephrectomy, years*			
< 40	1 (0.8)	8 (3.1)	9 (2.4)
≥ 40 and < 50	10 (8.3)	23 (8.8)	33 (8.7)
≥ 50 and < 60	29 (24.2)	49 (18.8)	78 (20.5)
≥ 60 and < 70	44 (36.7)	109 (41.8)	153 (40.2)
≥ 70	36 (30.0)	72 (27.6)	108 (28.3)
*Stage at initial diagnosis*			
I	27 (22.5)	84 (32.2)	111 (29.1)
II	17 (14.2)	35 (13.4)	52 (13.6)
III	58 (48.3)	111 (42.5)	169 (44.4)
IV	13 (10.8)	16 (6.1)	29 (7.6)
Unknown	5 (4.2)	15 (5.7)	20 (5.2)
**Perioperative treatment**			
*Neoadjuvant*			
Yes	3 (2.5)	7 (2.7)	10 (2.6)
No	117 (97.5)	253 (96.9)	370 (97.1)
Unknown	0	1 (0.4)	1 (0.3)
*Adjuvant*			
Yes	10 (8.3)	11 (4.2)	21 (5.5)
No	110 (91.7)	249 (95.4)	359 (94.2)
Unknown	0	1 (0.4)	1 (0.3)
*Objective for surgery other than primary site*			
Metastasectomy	17 (14.2)	44 (16.9)	61 (16.0)
Synchronous	1 (0.8)	2 (0.8)	3 (0.8)
Asynchronous	16 (13.3)	42 (16.1)	58 (15.2)
Others	1 (0.8)	9 (3.4)	10 (2.6)
**Pathological characteristics**			
*Immune phenotype*			
Excluded	99 (82.5)	73 (28.0)	172 (45.1)
Inflamed	17 (14.2)	3 (1.1)	20 (5.2)
Desert	4 (3.3)	185 (70.9)	189 (49.6)
Indeterminable	0	0	0
*Histology*			
Clear cell RCC	112 (93.3)	230 (88.1)	342 (89.8)
Papillary RCC	2 (1.7)	15 (5.7)	17 (4.5)
Chromophobe RCC	1 (0.8)	5 (1.9)	6 (1.6)
Spindle cell carcinoma	1 (0.8)	1 (0.4)	2 (0.5)
Others	4 (3.3)	10 (3.8)	14 (3.7)
Indeterminable	0	0	0
*Fuhrman grade*			
1	0	0	0
2	22 (18.3)	128 (49.0)	150 (39.4)
3	70 (58.3)	116 (44.4)	186 (48.8)
4	27 (22.5)	16 (6.1)	43 (11.3)
Indeterminable	1 (0.8)	1 (0.4)	2 (0.5)
*WHO/ISUP grade*			
1	0	0	0
2	31 (25.8)	147 (56.3)	178 (46.7)
3	51 (42.5)	91 (34.9)	142 (37.3)
4	37 (30.8)	22 (8.4)	59 (15.5)
Indeterminable	1 (0.8)	1 (0.4)	2 (0.5)
*Necrosis*			
Present	62 (51.7)	71 (27.2)	133 (34.9)
Absent	58 (48.3)	188 (72.0)	246 (64.6)
Indeterminable	0	2 (0.8)	2 (0.5)
*Vascular invasion*			
Present	27 (22.5)	50 (19.2)	77 (20.2)
Absent	85 (70.8)	199 (76.2)	284 (74.5)
Indeterminable	8 (6.7)	12 (4.6)	20 (5.2)
*Sarcomatoid component*			
Present	18 (15.0)	10 (3.8)	28 (7.3)
Absent	102 (85.0)	251 (96.2)	353 (92.7)
Indeterminable	0	0	0
*Growth pattern*			
Expansive	45 (37.5)	111 (42.5)	156 (40.9)
Infiltrative	31 (25.8)	52 (19.9)	83 (21.8)
Indeterminable	44 (36.7)	98 (37.5)	142 (37.3)

*IC*, tumor-infiltrating immune cells; *PD-L1*, programmed death-ligand 1; *RCC*, renal cell carcinoma; *WHO/ISUP*, World Health Organization/International Society of Urological Pathology

^a^Defined as IC1/2/3

^b^Defined as IC0

Clinical stage distribution at the time of diagnosis was slightly different between PD-L1-positive and -negative patients. In the PD-L1-positive group, 27 patients (22.5%) were in stage I, 17 (14.2%) were in stage II, 58 (48.3%) were in stage III, and 13 (10.8%) were in stage IV. In the PD-L1-negative group, 84 (32.2%), 35 (13.4%), 111 (42.5%), and 16 patients (6.1%) were in stages I to IV, respectively.

Distribution of some pathological features were imbalanced, with poor prognostic pathological features being more prevalent in the PD-L1-positive (Fuhrman grade 2: *n* = 22 [18.3%]; grade 3: *n* = 70 [58.3%]; grade 4: *n* = 27 [22.5%]) than in the PD-L1-negative group (Fuhrman grade 2: *n* = 128 [49.0%]; grade 3: *n* = 116 [44.4%]; grade 4: *n* = 16 [6.1%]). Similar trends were observed in WHO/ISUP grade distribution. Necrosis and sarcomatoid components were present in 62 (51.7%) and 18 (15.0%) PD-L1-positive patients and 71 (27.2%) and 10 (3.8%) PD-L1-negative patients, respectively.

Few patients received neoadjuvant or adjuvant therapy—10 (2.6%) and 21 (5.5%), respectively. Sunitinib was the most common neoadjuvant therapy (5 of 10 patients), whereas interferon alpha was the most common adjuvant therapy (14 of 21 patients; Online Resource 1). Metastasectomy was conducted in 61 patients (16.0%). Three patients underwent synchronous metastasectomy, and 58 had asynchronous metastasectomy. Clinicopathological characteristics at the time of first-line therapy are shown in Online Resource 2.

Patients were classified as high risk (*n* = 201), low risk (*n* = 160), and indeterminable (*n* = 20) based on clinical stage and Fuhrman grade (Online Resource 3). Although subgroups were defined based on both categories, most patients in stage I/II were categorized as low risk (only 3 patients [0.8%] were classified as high risk), and all patients in stage III/IV were categorized as high risk. More high- (36.3%) than low-risk patients (26.3%) were PD-L1 positive (Online Resource 4).

Baseline characteristics of patients by PD-L1 status and risk level are presented in Online Resource 5.

### Survival analysis by PD-L1 status

**Fig. 1:**
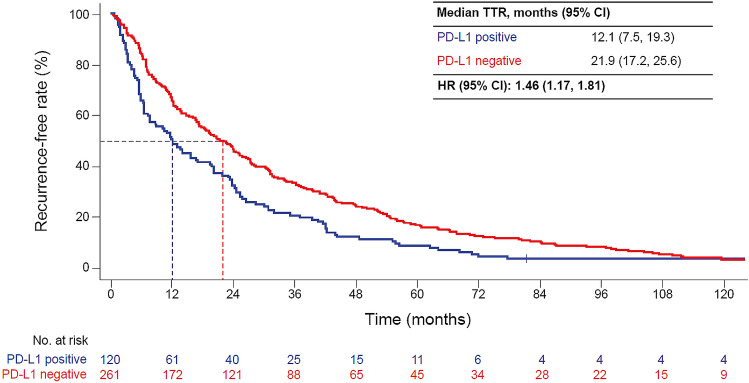
Time to recurrence from the time of nephrectomy by PD-L1 status. *HR*, hazard ratio; *PD-L1*, programmed death-ligand 1; *TTR*, time to recurrence

**Fig. 2:**
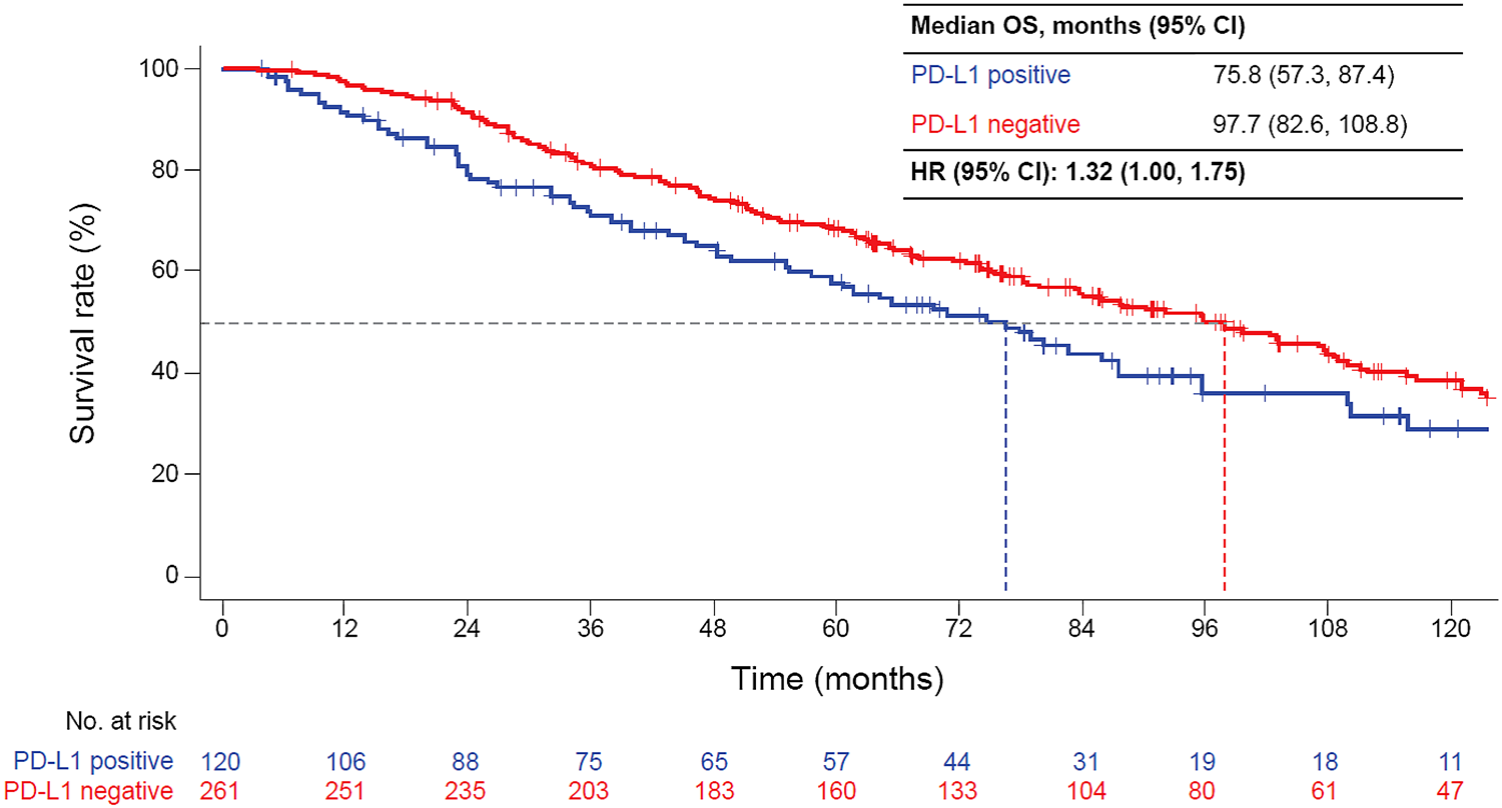
OS after nephrectomy by PD-L1 status. *HR*, hazard ratio; *OS*, overall survival; *PD-L1*, programmed death-ligand 1

Both TTR and OS were shorter in PD-L1-positive than -negative patients (mTTR 12.1 vs. 21.9 months [HR 1.46; 95% CI 1.17, 1.81]; mOS, 75.8 vs. 97.7 months [HR 1.32; 95% CI 1.00, 1.75]; Figs. 1, 2).

### Survival analysis by PD-L1 status and risk level

**Fig. 3:**
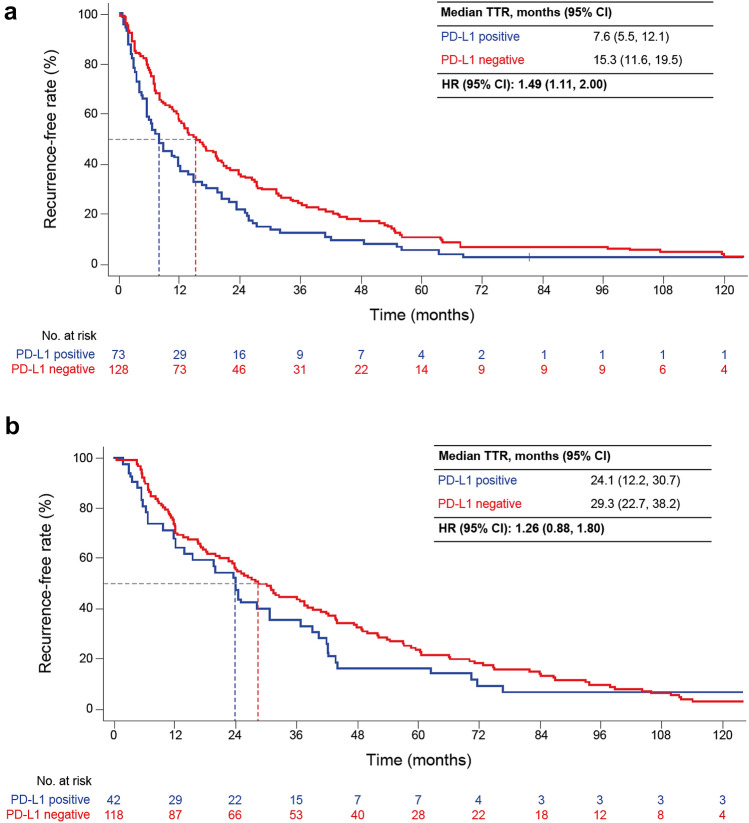
TTR from the time of nephrectomy by PD-L1 status in **a** high- and **b** low-risk patients. High risk was defined as stage III/IV or stage II and Fuhrman grade 4 at initial diagnosis. Low risk was defined as stage I or stage II and Fuhrman grade ≤ 3 at initial diagnosis. *HR*, hazard ratio; *PD-L1*, programmed death-ligand 1; *TTR*, time to recurrence

**Fig. 4:**
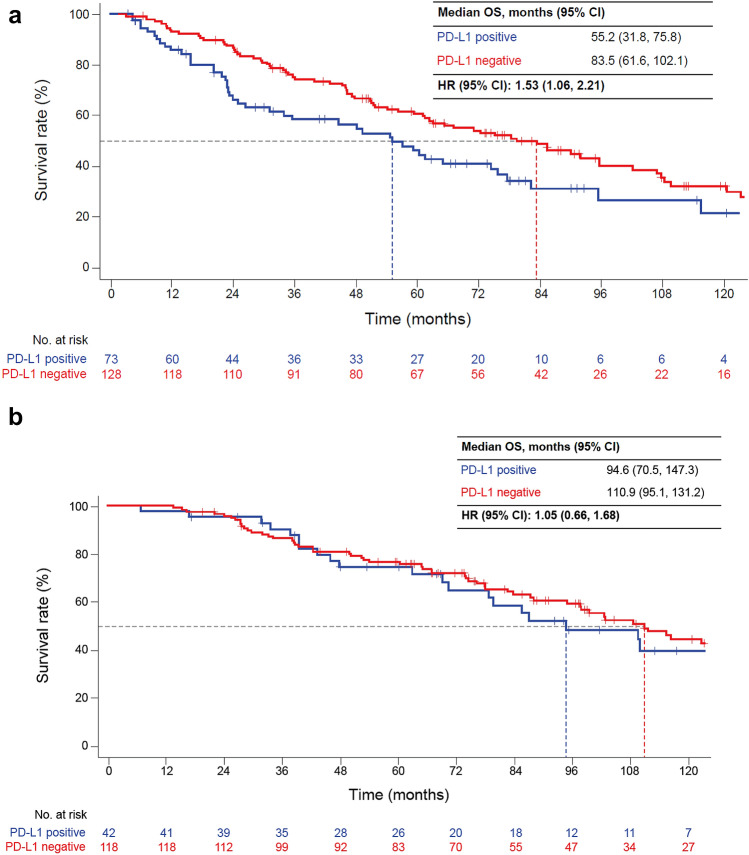
OS after nephrectomy by PD-L1 status in **a** high- and **b** low-risk patients. High risk was defined as stage III/IV or stage II and Fuhrman grade 4 at initial diagnosis. Low risk was defined as stage I or stage II and Fuhrman grade ≤ 3 at initial diagnosis. *HR*, hazard ratio; *OS*, overall survival; *PD-L1*, programmed death-ligand 1

In both high- and low-risk groups, TTR was shorter in PD-L1-positive than -negative patients (high risk: mTTR 7.6 vs. 15.3 months [HR 1.49, 95% CI 1.11, 2.00]; low risk: mTTR 24.1 vs. 29.3 months [HR 1.26; 95% CI 0.88, 1.80]; Fig. 3). OS was shorter in PD-L1-positive than -negative patients at high (mOS, 55.2 vs. 83.5 months; HR 1.53; 95% CI 1.06, 2.21) but not low risk (mOS, 94.6 vs. 110.9 months; HR 1.05; 95% CI 0.66, 1.68; Fig. 4).

### Survival analysis by PD-L1 status and nuclear grade

The difference in TTR between PD-L1-positive and -negative patients was observed only in those with Fuhrman grade 3 (HR 1.38; 95% CI 1.03, 1.87; Table 2) and WHO/ISUP grade 3 tumors (HR 1.37; 95% CI 0.97, 1.93; Online Resource 6). There were no differences in OS by PD-L1 status for any nuclear grade.

**Table 2:** Subgroup analysis by Fuhrman grade and PD-L1 status

Subgroup	Fuhrman grade	TTR					OS				
		PD-L1 positive^a^		PD-L1 negative^b^		HR (95% CI)	PD-L1 positive^a^		PD-L1 negative^b^		HR (95% CI)
		*n* (event)	Median (95% CI), months	*n* (event)	Median (95% CI), months		*n* (event)	Median (95% CI), months	*n* (event)	Median (95% CI), months	
**All**^**c**^	**1/2**	22 (22)	24.9 (11.9, 40.5)	128 (128)	28.1 (23.5, 34.2)	1.12 (0.71, 1.77)	22 (12)	110.0 (70.5, 206.1)	128 (65)	120.5 (102.8, 158.8)	1.13 (0.61, 2.09)
	**3**	70 (70)	11.9 (6.3, 19.3)	116 (116)	17.1 (12.2, 24.2)	1.38 (1.03, 1.87)	70 (42)	74.5 (54.9, 94.6)	116 (80)	76.4 (61.7, 95.6)	1.07 (0.73, 1.56)
	**4**	27 (26)	7.2 (4.7, 15.5)	16 (16)	10.8 (3.0, 20.6)	1.11 (0.59, 2.11)	27 (17)	49.4 (20.4, 75.8)	16 (13)	47.4 (24.2, 61.6)	0.88 (0.42, 1.83)
**High risk**	**1/2**	8 (8)	21.0 (2.3, 26.4)	53 (53)	23.6 (15.4, 32.1)	1.78 (0.83, 3.84)	8 (4)	105.5 (7.5, NE)	53 (25)	152.6 (90.0, 172.6)	1.52 (0.52, 4.47)
	**3**	42 (42)	9.6 (4.4, 13.5)	62 (62)	12.0 (7.8, 17.1)	1.28 (0.86, 1.90)	42 (28)	55.2 (26.6, 77.6)	62 (45)	63.0 (46.4, 83.5)	1.21 (0.75, 1.95)
	**4**	22 (21)	6.3 (3.4, 10.8)	13 (13)	7.0 (2.9, 19.4)	0.92 (0.46, 1.87)	22 (13)	48.2 (19.7, 130.9)	13 (11)	46.7 (17.9, 56.3)	0.73 (0.32, 1.68)
**Low risk**	**1/2**	13 (13)	30.7 (11.9, 62.3)	68 (68)	33.8 (23.8, 42.2)	0.99 (0.54, 1.80)	13 (7)	110.0 (46.1, 206.1)	68 (36)	110.9 (97.9, 161.9)	1.02 (0.45, 2.32)
	**3**	24 (24)	23.8 (6.3, 30.7)	46 (46)	24.6 (12.3, 41.9)	1.32 (0.79, 2.19)	24 (13)	94.6 (69.3, 147.3)	46 (29)	111.7 (65.1, 131.2)	0.91 (0.47, 1.75)
	**4**	5 (5)	15.6 (4.7, 70.6)	3 (3)	57.3 (11.8, 104.2)	2.18 (0.41, 11.62)	5 (4)	63.1 (7.0, 194.4)	3 (2)	78.1 (24.2, NE)	1.30 (0.21, 8.20)

*HR*, hazard ratio; *IC*, tumor-infiltrating immune cells; *NE*, not evaluable; *OS*, overall survival; *PD-L1*, programmed death-ligand 1; *TTR*, time to recurrence

^a^Defined as IC1/2/3

^b^Defined as IC0

^c^All patients with a known Fuhrman grade (*n* = 379) were included in this analysis

## Discussion

### Clinical application in adjuvant therapy

In this retrospective ARCHERY secondary analysis, PD-L1-positive patients had shorter TTR and OS than PD-L1-negative patients in all recurrent cases. Furthermore, in the high-risk group, but not in the low-risk group, TTR and OS were shorter in PD-L1-positive versus -negative patients.

Although several vascular endothelial growth factor receptor-targeted therapies showed improved clinical outcomes in metastatic RCC, a meta-analysis of phase 3 RCTs showed that adjuvant vascular endothelial growth factor receptor-targeted therapies had no benefit in patients with intermediate- or high-risk local or regional fully resected RCC [12]. Thus, a significant unmet medical need for an efficacious, well-tolerated adjuvant therapy for reducing RCC recurrence risk remains. Several ongoing phase 3 RCTs are examining the effect of immune CPIs, including PD-(L)1 inhibitors for high-risk locally or regionally resected RCC [6]. KEYNOTE-564 showed significant improvement in disease-free survival of patients with localized RCC who received pembrolizumab as adjuvant therapy [7]. Therefore, PD-L1-positive patients who have high recurrence risk in this ARCHERY secondary analysis can potentially benefit from adjuvant treatment with CPIs.

### TTR and OS difference between PD-L1-positive and -negative patients

Compared with the PD-L1-negative group, more PD-L1-positive patients had poor prognostic pathological features (Fuhrman grade, WHO/ISUP grade, necrosis, and sarcomatoid components). This trend was consistent with another study that reported a higher percentage of patients with clear cell RCC who had high nuclear grades and tumor necrosis in the group with high aggregate intratumoral PD-L1 expression versus the group without [13]. Since nuclear grade has shown prognostic abilities in RCC-specific survival and was a covariate in the commonly used prognostic models for localized RCC [3, 14, 15], these results suggest that differences in TTR and OS between PD-L1-positive and -negative groups might be due to the imbalance in the number of patients with higher nuclear grade.

Differences in TTR between PD-L1-positive and -negative groups were observed only in the subgroups with grade 3 nuclear grade (both Fuhrman and WHO/ISUP grades). The small number of patients with grade 4 nuclear grade could have affected the accuracy of TTR estimation. These results suggest a possible relationship between nuclear grade and PD-L1 expression and should be considered in prognostic risk evaluation.

In contrast, OS results in each nuclear grade consistently showed no difference between PD-L1-positive and -negative patients. Since all patients in this study received systemic therapy after recurrence, the impact of systemic treatment should be considered in the interpretation of OS results. A trial-level meta-analysis reported only a modest correlation (*R*^2^ = 0.48; 95% CI 0.14, 0.67) between 5‐year disease-free survival and 5‐year OS rates in localized RCC [16], suggesting that further investigation is required.

### TTR and OS difference between PD-L1-positive and -negative patients in the high-risk and low-risk subgroups

Both TTR and OS were shorter in PD-L1-positive versus -negative patients in the high-risk subgroup. Although more patients had poor prognostic pathological features (Fuhrman grade, WHO/ISUP grade, necrosis, and sarcomatoid components) in the high-risk PD-L1-positive versus -negative group (Online Resource 5), differences in TTR and OS of high-risk patients between PD-L1-positive and -negative groups were observed in each nuclear grade (both Fuhrman and WHO/ISUP grades; Table 2, Online Resource 6).

In the low-risk group, TTR was shorter in PD-L1-positive versus -negative patients, whereas OS was similar in both PD-L1-positive and -negative patients. Differences in TTR of low-risk patients between PD-L1-positive and -negative groups were also observed in each nuclear grade. However, differences in OS between low-risk PD-L1-positive and -negative patients were not observed in Grade 1/2 and Grade 3 subgroups; the Grade 4 subgroup was limited by the number of patients (Table 2, Online Resource 6).

Although there is currently no definitive explanation for the different trends in TTR and OS observed between high-risk and low-risk patients, it is noteworthy that patients who are recurrent-free—i.e., those who form the majority of the low-risk population—were not included in this study. The trend of shorter TTR and OS in high-risk patients who were PD-L1 positive versus negative, which was also seen within each nuclear grade subgroup, highlights the importance of PD-L1 as a target for adjuvant therapy.

### Limitations

ARCHERY enrolled patients with RCC who received systemic therapy for metastatic or recurrent disease. Hence, this secondary analysis included only those with recurrent RCC. Partial or radical nephrectomy is curative in the majority of the patients, with approximately 30% of patients developing disease recurrence [17]. Considering that recurrence-free cases were excluded in this analysis, the generalizability of these findings is limited to a subset of high-risk patients.

Currently, there is no standard therapy established in Japan, and most of the patients analyzed in this analysis did not receive any post-nephrectomy treatment. Hence, the prognostic impact of PD-L1 in this analysis is not reflective of the response to CPIs after nephrectomy in patients with PD-L1-positive RCC. It is noteworthy that longer OS was observed in PD-L1-positive patients who received CPIs after second-line therapy in ARCHERY [8]. However, further analysis on progression-free survival may provide more insight into the prognostic impact of PD-L1.

Due to the retrospective nature of ARCHERY, there might be residual confounding bias in the subgroups stratified by nuclear grade alone. There is also a limitation in comparing baseline clinicopathological characteristics due to the small number of patients within each category by risk and PD-L1 status.

The risk levels of patients were determined based on clinical stage, and not the TNM T stage that is incorporated in the most used risk classification models. Information on TNM staging was not collected because clinical stage was considered sufficient, given that the ARCHERY study focused on OS after 1L treatment of recurrent or metastatic RCC. Furthermore, this study included radical or partial nephrectomy conducted in 2009 and before and in 2016 and after. Hence, the clinical stage at initial diagnosis was based on different editions of TNM. Twenty patients (grade 2: *n* = 8; grade 3; *n* = 12) were excluded due to the lack of clinical stage information. Information on whether patients underwent partial or radical nephrectomy was also not collected. Fuhrman grade, instead of WHO/ISUP grade, was selected for defining high- and low-risk subgroups based on current prediction models such as UISS and MSKCC [3]. However, international pathological protocols have replaced Fuhrman grade with WHO/ISUP grade [18–20]. A change in nuclear grading system would affect the classification of patients, particularly in stage II.

## Conclusions

This secondary analysis of ARCHERY provided insight to the association between PD-L1 expression and nuclear grade, two widely known prognostic factors, thereby improving our understanding of RCC prognosis. These results suggest that PD-L1 expression may play a role in predicting the OS and risk of recurrence in high-risk patients. However, the influence of nuclear grade should also be considered.

## Supplementary Information

Below is the link to the electronic supplementary material.

Supplementary file 1 (DOCX 58 KB)

## Data Availability

Qualified researchers may request access to individual patient-level data through the clinical study data request platform (www.clinicalstudydatarequest.com). For further details on Chugai’s Data Sharing Policy and how to request access to related clinical study documents, see www.chugai-pharm.co.jp/english/profile/rd/ctds_request.html.
