# Supplementary material for: Prognostic value of PD-L1 expression in recurrent renal cell carcinoma after nephrectomy: a secondary analysis of the ARCHERY study
Source: Int J Clin Oncol. 2022 Dec 19;28(2):289–98. doi: 10.1007/s10147-022-02256-z (PMC9889451; doi:10.1007/s10147-022-02256-z)
Supplement: Supplementary file 1 — (DOCX 58 KB) [file 10147_2022_2256_MOESM1_ESM.docx]

**ONLINE RESOURCES**

**Prognostic value of PD-L1 expression in recurrent renal cell carcinoma after nephrectomy: a secondary analysis of the ARCHERY study**

*International Journal of Clinical Oncology*

Satoshi Tamada, Masahiro Nozawa, Kojiro Ohba, Ryuichi Mizuno, Atsushi Takamoto, Chisato Ohe, Takuya Yoshimoto, Yuki Nakagawa, Tamaki Fukuyama, Nobuaki Matsubara, Go Kimura, Yoshihiko Tomita, Norio Nonomura, Masatoshi Eto

**Corresponding author**

Norio Nonomura | https://orcid.org/0000-0002-6522-6233

Department of Urology, Osaka University Graduate School of Medicine, 2-2 Yamadaoka, Suita, Osaka, 565-0871, Japan

Tel: +81-6-6879-3534

Fax: +81-6-6879-3539

Email: [nono@uro.med.osaka-u.ac.jp](mailto:nono@uro.med.osaka-u.ac.jp)

**Online Resource 1 Perioperative treatment by PD-L1 status**

| **Perioperative treatment, n (%)** | **PD-L1 positive^a^  (n=120)** | **PD-L1 negative^b^**  **(n=261)** | **Total  (N=381)** |
| --- | --- | --- | --- |
| **Neoadjuvant treatment** |  |  |  |
| Sunitinib | 2 (1.7) | 3 (1.1) | 5 (1.3) |
| Axitinib | 1 (0.8) | 2 (0.8) | 3 (0.8) |
| Interferon alpha | 0 | 1 (0.4) | 1 (0.3) |
| Sorafenib | 0 | 1 (0.4) | 1 (0.3) |
| Pazopanib | 0 | 1 (0.4) | 1 (0.3) |
| **Adjuvant treatment** |  |  |  |
| Interferon alpha | 6 (5.0) | 8 (3.1) | 14 (3.7) |
| Sunitinib | 2 (1.7) | 0 | 2 (0.5) |
| Axitinib | 1 (0.8) | 3 (1.1) | 4 (1.0) |
| Pazopanib | 1 (0.8) | 0 | 1 (0.3) |
| **Resected organ by metastasectomy (≥3%)** |  |  |  |
| Lung | 8 (6.7) | 22 (8.4) | 30 (7.9) |
| Lymph node | 5 (4.2) | 3 (1.1) | 8 (2.1) |
| Pancreas | 4 (3.3) | 1 (0.4) | 5 (1.3) |
| Bone | 0 | 12 (4.6) | 12 (3.1) |
| IC, tumor-infiltrating immune cells; PD-L1, programmed death-ligand 1.  ^a^ Defined as IC1/2/3.  ^b^ Defined as IC0. | | | |

**Online Resource 2 Clinicopathological characteristics at time of 1L therapy of patients who underwent nephrectomy by PD-L1 status**

| **Characteristics, n (%)** | **PD-L1 positive^a^  (n=120)** | **PD-L1 negative^b^**  **(n=261)** | **Total  (N=381)** |
| --- | --- | --- | --- |
| **Time from diagnosis to 1L therapy, years** |  |  |  |
| ≤2 | 71 (59.2) | 109 (41.8) | 180 (47.2) |
| >2 | 49 (40.8) | 152 (58.2) | 201 (52.8) |
| **MSKCC risk** |  |  |  |
| Favorable | 33 (27.5) | 121 (46.4) | 154 (40.4) |
| Intermediate | 76 (63.3) | 130 (49.8) | 206 (54.1) |
| Poor | 11 (9.2) | 10 (3.8) | 21 (5.5) |
| **IMDC risk** |  |  |  |
| Favorable | 31 (25.8) | 109 (41.8) | 140 (36.7) |
| Intermediate | 66 (55.0) | 132 (50.6) | 198 (52.0) |
| Poor | 23 (19.2) | 20 (7.7) | 43 (11.3) |
| **Distant metastases** |  |  |  |
| Present | 112 (93.3) | 247 (94.6) | 359 (94.2) |
| Absent | 8 (6.7) | 11 (4.2) | 19 (5.0) |
| Unknown | 0 | 3 (1.1) | 3 (0.8) |
| **Metastatic site (≥3%)** |  |  |  |
| Lung | 73 (60.8) | 157 (60.2) | 230 (60.4) |
| Lymph node | 26 (21.7) | 44 (16.9) | 70 (18.4) |
| Bone | 20 (16.7) | 57 (21.8) | 77 (20.2) |
| Liver | 15 (12.5) | 28 (10.7) | 43 (11.3) |
| Pancreas | 8 (6.7) | 12 (4.6) | 20 (5.2) |
| Kidney (recurrent) | 7 (5.8) | 8 (3.1) | 15 (3.9) |
| Pleura | 7 (5.8) | 5 (1.9) | 12 (3.1) |
| Adrenal gland | 6 (5.0) | 14 (5.4) | 20 (5.2) |
| Soft tissue | 5 (4.2) | 7 (2.7) | 12 (3.1) |
| Peritoneum | 2 (1.7) | 9 (3.4) | 11 (2.9) |
| 1L, first line; IC, tumor-infiltrating immune cells; IMDC, International Metastatic RCC Database Consortium; MSKCC, Memorial Sloan Kettering Cancer Center; PD-L1, programmed death-ligand 1.  ^a^ Defined as IC1/2/3.  ^b^ Defined as IC0. | | | |

**Online Resource 3 Cross table of clinical stage and Fuhrman grade^a^**

| **Stage at initial diagnosis, n (%)** | **Fuhrman grade^b^** | | | | **Total** |
| --- | --- | --- | --- | --- | --- |
|  | **2** | **3** | **4** | **Indeterminable** |  |
| I | 57 (15.0) | 45 (11.8) | 8 (2.1) | 1 (0.3) | 111 (29.1) |
| II | 24 (6.3) | 25 (6.6) | 3 (0.8) | 0 | 52 (13.6) |
| III | 55 (14.4) | 88 (23.1) | 26 (6.8) | 0 | 169 (44.4) |
| IV | 6 (1.6) | 16 (4.2) | 6 (1.6) | 1 (0.3) | 29 (7.6) |
| Unknown | 8 (2.1) | 12 (3.1) | 0 | 0 | 20 (5.2) |
| Total | 150 (39.4) | 186 (48.8) | 43 (11.3) | 2 (0.5) | 381 |
| ^a^ Patients who were in the blue, pink, and gray categories were classified as low risk, high risk, and not determined, respectively.  ^b^ There were no patients classified as Fuhrman grade 1. | | | | | |

**Online Resource 4 PD-L1 expression by subgroups**

| **PD-L1 expression, n (%)** | **Recurrent**  **(N=381)** | **Recurrent  (high risk^a^)**  **(n=201)** | **Recurrent  (low risk^b^)**  **(n=160)** |
| --- | --- | --- | --- |
| IC0 | 261 (68.5) | 128 (63.7) | 118 (73.8) |
| IC1 | 80 (21.0) | 47 (23.4) | 29 (18.1) |
| IC2 | 20 (5.2) | 10 (5.0) | 10 (6.3) |
| IC3 | 20 (5.2) | 16 (8.0) | 3 (1.9) |
|  |  |  |  |
| Negative^c^ | 261 (68.5) | 128 (63.7) | 118 (73.8) |
| Positive^d^ | 120 (31.5) | 73 (36.3) | 42 (26.3) |
| IC, tumor-infiltrating immune cells; PD-L1, programmed death-ligand 1.  ^a^ Defined as stage III/IV or stage II and Fuhrman grade 4 at initial diagnosis. ^b^ Defined as stage I or stage II and Fuhrman grade ≤3 at initial diagnosis. ^c^ Defined as IC0.  ^d^ Defined as IC1/2/3. | | | |

**Online Resource 5 Clinicopathological characteristics of patients who underwent nephrectomy by PD-L1 status and risk level**

| **Characteristics, n (%)** | **High risk^a^** | | | **Low risk^b^** | | |
| --- | --- | --- | --- | --- | --- | --- |
|  | **PD-L1**  **positive^c^  (n=73)** | **PD-L1**  **negative^d^  (n=128)** | **Total^e^  (n=201)** | **PD-L1 positive^c^  (n=42)** | **PD-L1 negative^d^  (n=118)** | **Total^e^  (n=160)** |
| **At initial diagnosis or nephrectomy** |  |  |  |  |  |  |
| **Male** | 53 (72.6) | 91 (71.1) | 144 (71.6) | 31 (73.8) | 97 (82.2) | 128 (80.0) |
| **Female** | 20 (27.4) | 37 (28.9) | 57 (28.4) | 11 (26.2) | 21 (17.8) | 32 (20.0) |
| **Age, years** |  |  |  |  |  |  |
| <40 | 1 (1.4) | 6 (4.7) | 7 (3.5) | 0 | 2 (1.7) | 2 (1.3) |
| ≥40 and <50 | 6 (8.2) | 9 (7.0) | 15 (7.5) | 4 (9.5) | 12 (10.2) | 16 (10.0) |
| ≥50 and <60 | 16 (21.9) | 23 (18.0) | 39 (19.4) | 12 (28.6) | 23 (19.5) | 35 (21.9) |
| ≥60 and <70 | 28 (38.4) | 55 (43.0) | 83 (41.3) | 15 (35.7) | 47 (39.8) | 62 (38.8) |
| ≥70 | 22 (30.1) | 35 (27.3) | 57 (28.4) | 11 (26.2) | 34 (28.8) | 45 (28.1) |
| **Stage at initial diagnosis** |  |  |  |  |  |  |
| I | 0 | 0 | 0 | 27 (64.3) | 84 (71.2) | 111 (69.4) |
| II | 2 (2.7) | 1 (0.8) | 3 (1.5) | 15 (35.7) | 34 (28.8) | 49 (30.6) |
| III | 58 (79.5) | 111 (86.7) | 169 (84.1) | 0 | 0 | 0 |
| IV | 13 (17.8) | 16 (12.5) | 29 (14.4) | 0 | 0 | 0 |
| Unknown | 0 | 0 | 0 | 0 | 0 | 0 |
| **Perioperative treatment** |  |  |  |  |  |  |
| **Neoadjuvant** |  |  |  |  |  |  |
| Yes | 3 (4.1) | 7 (5.5) | 10 (5.0) | 0 | 0 | 0 |
| No | 70 (95.9) | 121 (94.5) | 191 (95.0) | 42 (100.0) | 117 (99.2) | 159 (99.4) |
| Unknown | 0 | 0 | 0 | 0 | 1 (0.8) | 1 (0.6) |
| **Treatment for neoadjuvant** |  |  |  |  |  |  |
| Axitinib | 1 (1.4) | 2 (1.6) | 3 (1.5) | 0 | 0 | 0 |
| Interferon alpha | 0 | 1 (0.8) | 1 (0.5) | 0 | 0 | 0 |
| Sunitinib | 2 (2.7) | 3 (2.3) | 5 (2.5) | 0 | 0 | 0 |
| Sorafenib | 0 | 1 (0.8) | 1 (0.5) | 0 | 0 | 0 |
| Pazopanib | 0 | 1 (0.8) | 1 (0.5) | 0 | 0 | 0 |
| **Adjuvant** |  |  |  |  |  |  |
| Yes | 9 (12.3) | 7 (5.5) | 16 (8.0) | 1 (2.4) | 4 (3.4) | 5 (3.1) |
| No | 64 (87.7) | 121 (94.5) | 185 (92.0) | 41 (97.6) | 113 (95.8) | 154 (96.3) |
| Unknown | 0 | 0 | 0 | 0 | 1 (0.8) | 1 (0.6) |
| **Treatment for adjuvant** |  |  |  |  |  |  |
| Axitinib | 1 (1.4) | 3 (2.3) | 4 (2.0) | 0 | 0 | 0 |
| Interferon alpha | 5 (6.8) | 4 (3.1) | 9 (4.5) | 1 (2.4) | 4 (3.4) | 5 (3.1) |
| Sunitinib | 2 (2.7) | 0 | 2 (1.0) | 0 | 0 | 0 |
| Pazopanib | 1 (1.4) | 0 | 1 (0.5) | 0 | 0 | 0 |
| **Resected organ by metastasectomy (≥3%)** |  |  |  |  |  |  |
| Lung | 5 (6.8) | 3 (2.3) | 8 (4.0) | 3 (7.1) | 15 (12.7) | 18 (11.3) |
| Lymph node | 3 (4.1) | 0 | 3 (1.5) | 2 (4.8) | 3 (2.5) | 5 (3.1) |
| Bone | 0 | 5 (3.9) | 5 (2.5) | 0 | 6 (5.1) | 6 (3.8) |
| **Objective for surgery other than primary site** |  |  |  |  |  |  |
| Metastasectomy | 8 (11.0) | 12 (9.4) | 20 (10.0) | 8 (19.0) | 25 (21.2) | 33 (20.6) |
| Others | 1 (1.4) | 4 (3.1) | 5 (2.5) | 0 | 5 (4.2) | 5 (3.1) |
| **At the time of 1L therapy** |  |  |  |  |  |  |
| **Time from initial diagnosis to 1L therapy, years** |  |  |  |  |  |  |
| ≤2 | 53 (72.6) | 67 (52.3) | 120 (59.7) | 16 (38.1) | 39 (33.1) | 55 (34.4) |
| >2 | 20 (27.4) | 61 (47.7) | 81 (40.3) | 26 (61.9) | 79 (66.9) | 105 (65.6) |
| **MSKCC risk** |  |  |  |  |  |  |
| Favorable | 17 (23.3) | 52 (40.6) | 69 (34.3) | 13 (31.0) | 64 (54.2) | 77 (48.1) |
| Intermediate | 46 (63.0) | 69 (53.9) | 115 (57.2) | 28 (66.7) | 51 (43.2) | 79 (49.4) |
| Poor | 10 (13.7) | 7 (5.5) | 17 (8.5) | 1 (2.4) | 3 (2.5) | 4 (2.5) |
| **IMDC risk** |  |  |  |  |  |  |
| Favorable | 17 (23.3) | 48 (37.5) | 65 (32.3) | 11 (26.2) | 57 (48.3) | 68 (42.5) |
| Intermediate | 39 (53.4) | 66 (51.6) | 105 (52.2) | 25 (59.5) | 55 (46.6) | 80 (50.0) |
| Poor | 17 (23.3) | 14 (10.9) | 31 (15.4) | 6 (14.3) | 6 (5.1) | 12 (7.5) |
| **Distant metastases** |  |  |  |  |  |  |
| Present | 67 (91.8) | 122 (95.3) | 189 (94.0) | 40 (95.2) | 112 (94.9) | 152 (95.0) |
| Absent | 6 (8.2) | 5 (3.9) | 11 (5.5) | 2 (4.8) | 4 (3.4) | 6 (3.8) |
| Unknown | 0 | 1 (0.8) | 1 (0.5) | 0 | 2 (1.7) | 2 (1.3) |
| **Metastatic site (≥3%)** |  |  |  |  |  |  |
| Lung | 46 (63.0) | 76 (59.4) | 122 (60.7) | 24 (57.1) | 71 (60.2) | 95 (59.4) |
| Lymph node | 20 (27.4) | 26 (20.3) | 46 (22.9) | 6 (14.3) | 17 (14.4) | 23 (14.4) |
| Bone | 12 (16.4) | 24 (18.8) | 36 (17.9) | 8 (19.0) | 32 (27.1) | 40 (25.0) |
| Liver | 12 (16.4) | 20 (15.6) | 32 (15.9) | 2 (4.8) | 8 (6.8) | 10 (6.3) |
| Adrenal gland | 5 (6.8) | 4 (3.1) | 9 (4.5) | 1 (2.4) | 10 (8.5) | 11 (6.9) |
| Soft tissue | 5 (6.8) | 4 (3.1) | 9 (4.5) | 0 | 3 (2.5) | 3 (1.9) |
| Kidney (recurrence) | 4 (5.5) | 3 (2.3) | 7 (3.5) | 2 (4.8) | 3 (2.5) | 5 (3.1) |
| Pleura | 4 (5.5) | 2 (1.6) | 6 (3.0) | 3 (7.1) | 3 (2.5) | 6 (3.8) |
| Pancreas | 2 (2.7) | 6 (4.7) | 8 (4.0) | 6 (14.3) | 5 (4.2) | 11 (6.9) |
| Peritoneum | 1 (1.4) | 5 (3.9) | 6 (3.0) | 1 (2.4) | 4 (3.4) | 5 (3.1) |
| Brain | 1 (1.4) | 4 (3.1) | 5 (2.5) | 2 (4.8) | 1 (0.8) | 3 (1.9) |
| **Pathological characteristics** |  |  |  |  |  |  |
| **Immune phenotype** |  |  |  |  |  |  |
| Excluded | 61 (83.6) | 31 (24.2) | 92 (45.8) | 33 (78.6) | 38 (32.2) | 71 (44.4) |
| Inflamed | 9 (12.3) | 1 (0.8) | 10 (5.0) | 8 (19.0) | 2 (1.7) | 10 (6.3) |
| Desert | 3 (4.1) | 96 (75.0) | 99 (49.3) | 1 (2.4) | 78 (66.1) | 79 (49.4) |
| Indeterminable | 0 | 0 | 0 | 0 | 0 | 0 |
| **Histology** |  |  |  |  |  |  |
| Clear cell RCC | 68 (93.2) | 108 (84.4) | 176 (87.6) | 40 (95.2) | 108 (91.5) | 148 (92.5) |
| Papillary RCC | 1 (1.4) | 11 (8.6) | 12 (6.0) | 1 (2.4) | 4 (3.4) | 5 (3.1) |
| Chromophobe RCC | 1 (1.4) | 2 (1.6) | 3 (1.5) | 0 | 3 (2.5) | 3 (1.9) |
| Spindle cell carcinoma | 1 (1.4) | 1 (0.8) | 2 (1.0) | 0 | 0 | 0 |
| Others | 2 (2.7) | 6 (4.7) | 8 (4.0) | 1 (2.4) | 3 (2.5) | 4 (2.5) |
| Indeterminable | 0 | 0 | 0 | 0 | 0 | 0 |
| **Fuhrman grade** |  |  |  |  |  |  |
| 1 | 0 | 0 | 0 | 0 | 0 | 0 |
| 2 | 8 (11.0) | 53 (41.4) | 61 (30.3) | 13 (31.0) | 68 (57.6) | 81 (50.6) |
| 3 | 42 (57.5) | 62 (48.4) | 104 (51.7) | 24 (57.1) | 46 (39.0) | 70 (43.8) |
| 4 | 22 (30.1) | 13 (10.2) | 35 (17.4) | 5 (11.9) | 3 (2.5) | 8 (5.0) |
| Indeterminable | 1 (1.4) | 0 | 1 (0.5) | 0 | 1 (0.8) | 1 (0.6) |
| **WHO/ISUP grade** |  |  |  |  |  |  |
| 1 | 0 | 0 | 0 | 0 | 0 | 0 |
| 2 | 10 (13.7) | 63 (49.2) | 73 (36.3) | 17 (40.5) | 76 (64.4) | 93 (58.1) |
| 3 | 35 (47.9) | 49 (38.3) | 84 (41.8) | 15 (35.7) | 35 (29.7) | 50 (31.3) |
| 4 | 27 (37.0) | 16 (12.5) | 43 (21.4) | 10 (23.8) | 6 (5.1) | 16 (10.0) |
| Indeterminable | 1 (1.4) | 0 | 1 (0.5) | 0 | 1 (0.8) | 1 (0.6) |
| **Necrosis** |  |  |  |  |  |  |
| Present | 44 (60.3) | 46 (35.9) | 90 (44.8) | 18 (42.9) | 24 (20.3) | 42 (26.3) |
| Absent | 29 (39.7) | 80 (62.5) | 109 (54.2) | 24 (57.1) | 94 (79.7) | 118 (73.8) |
| Indeterminable | 0 | 2 (1.6) | 2 (1.0) | 0 | 0 | 0 |
| **Vascular invasion** |  |  |  |  |  |  |
| Present | 19 (26.0) | 34 (26.6) | 53 (26.4) | 6 (14.3) | 13 (11.0) | 19 (11.9) |
| Absent | 50 (68.5) | 86 (67.2) | 136 (67.7) | 32 (76.2) | 102 (86.4) | 134 (83.8) |
| Indeterminable | 4 (5.5) | 8 (6.3) | 12 (6.0) | 4 (9.5) | 3 (2.5) | 7 (4.4) |
| **Sarcomatoid component** |  |  |  |  |  |  |
| Present | 17 (23.3) | 8 (6.3) | 25 (12.4) | 1 (2.4) | 2 (1.7) | 3 (1.9) |
| Absent | 56 (76.7) | 120 (93.8) | 176 (87.6) | 41 (97.6) | 116 (98.3) | 157 (98.1) |
| Indeterminable | 0 | 0 | 0 | 0 | 0 | 0 |
| **Growth pattern** |  |  |  |  |  |  |
| Expansive | 20 (27.4) | 39 (30.5) | 59 (29.4) | 24 (57.1) | 67 (56.8) | 91 (56.9) |
| Infiltrative | 23 (31.5) | 36 (28.1) | 59 (29.4) | 7 (16.7) | 14 (11.9) | 21 (13.1) |
| Indeterminable | 30 (41.1) | 53 (41.4) | 83 (41.3) | 11 (26.2) | 37 (31.4) | 48 (30.0) |
| 1L, first line; IC, tumor-infiltrating immune cells; IMDC, International Metastatic RCC Database Consortium; MSKCC, Memorial Sloan Kettering Cancer Center; PD-L1, programmed death-ligand 1; RCC, renal cell carcinoma; WHO/ISUP, World Health Organization/International Society of Urological Pathology.  ^a^ Defined as stage III/IV or stage II and Fuhrman grade 4 at initial diagnosis. ^b^ Defined as stage I or stage II and Fuhrman grade ≤3 at initial diagnosis. ^c^ Defined as IC1/2/3.  ^d^ Defined as IC0.  ^e^ There were 20 patients who were not categorized as high or low risk due to unknown clinical stage (n=20) or indeterminable Fuhrman grade (n=2). | | | | | | |

**Online resource 6 Subgroup analysis by WHO/ISUP grade and PD-L1 status**

|  |  | **TTR** | | | | | **OS** | | | | |
| --- | --- | --- | --- | --- | --- | --- | --- | --- | --- | --- | --- |
|  |  | **PD-L1 positive^a^** | | **PD-L1 negative^b^** | | **HR  (95% CI)** | **PD-L1 positive^a^** | | **PD-L1 negative^b^** | | **HR  (95% CI)** |
| **Subgroup** | **WHO/ ISUP grade** | **n**  **(event)** | **Median (95% CI), months** | **n**  **(event)** | **Median  (95% CI), months** |  | **n**  **(event)** | **Median (95% CI), months** | **n**  **(event)** | **Median (95% CI), months** |  |
| **All^c^** | **1/2** | 31  (31) | 24.0  (13.9, 40.5) | 147  (147) | 28.3  (23.9, 33.8) | 1.16  (0.79, 1.71) | 31  (14) | 110.0  (85.7, 206.1) | 147  (78) | 110.9  (102.8, 145.3) | 0.93 (0.53, 1.65) |
|  | **3** | 51  (51) | 8.8  (5.7, 19.7) | 91  (91) | 15.1  (10.6, 20.6) | 1.37  (0.97, 1.93) | 51  (31) | 65.1  (43.4, 109.6) | 91  (63) | 66.8  (59.4, 85.0) | 1.04  (0.67, 1.60) |
|  | **4** | 37  (36) | 7.6  (5.3, 15.5) | 22  (22) | 12.2  (6.9, 20.1) | 1.09  (0.64, 1.88) | 37  (26) | 44.8  (23.9, 75.8) | 22  (17) | 54.3  (39.4, 78.1) | 1.13  (0.61, 2.10) |
| **High risk** | **1/2** | 10  (10) | 16.4  (2.3, 21.8) | 63  (63) | 24.9  (16.4, 32.1) | 2.07  (1.04, 4.14) | 10  (3) | 95.4  (7.5, NE) | 63  (32) | 109.6  (91.9, 161.1) | 1.02 (0.31, 3.40) |
|  | **3** | 35  (35) | 8.8  (4.4, 16.9) | 49  (49) | 10.6  (6.9, 15.1) | 1.06  (0.69, 1.64) | 35  (24) | 57.3  (26.6, 82.3) | 49  (36) | 51.4  (31.4, 79.4) | 0.99  (0.58, 1.67) |
|  | **4** | 27  (26) | 6.3  (3.4, 10.8) | 16  (16) | 9.4  (3.0, 14.7) | 1.05  (0.56, 1.98) | 27  (18) | 44.8  (19.7, 75.8) | 16  (13) | 54.3  (24.9, 61.6) | 0.99  (0.48, 2.07) |
| **Low risk** | **1/2** | 17  (17) | 40.5  (13.9, 62.3) | 76  (76) | 31.7  (23.9, 42.2) | 0.95  (0.56, 1.61) | 17  (10) | 110.0  (69.3, 206.1) | 76  (41) | 111.7  (97.9, 151.6) | 1.05 (0.52, 2.10) |
|  | **3** | 15  (15) | 9.7  (3.6, 24.1) | 35  (35) | 25.0  (12.1, 43.7) | 1.45  (0.77, 2.73) | 15  (6) | 109.6  (47.9, NE) | 35  (22) | 87.4  (65.1, 151.6) | 0.64  (0.26, 1.59) |
|  | **4** | 10  (10) | 20.4  (4.7, 36.8) | 6  (6) | 20.4  (8.2, 104.2) | 1.42  (0.48, 4.23) | 10  (8) | 63.1  (7.0, 87.2) | 6  (4) | 65.5  (24.2, NE) | 1.40  (0.41, 4.80) |

HR, hazard ratio; IC, tumor-infiltrating immune cells; NE, not evaluable; OS, overall survival; PD-L1, programmed death-ligand 1; TTR, time to recurrence; WHO/ISUP, World Health Organization/International Society of Urological Pathology.

^a^ Defined as IC1/2/3.

^b^ Defined as IC0.

^c^ All patients with a known Fuhrman grade (n=379) were included in this analysis.

**Online Resource 7 List of participating sites and investigators**

| **Site** | **Principal Investigator** |
| --- | --- |
| Hokkaido University Hospital | T. Osawa |
| Sapporo Medical University Hospital | N. Masumori |
| Hirosaki University Hospital | S. Hatakeyama |
| Iwate Medical University | W. Obara |
| University of Tsukuba Hospital | T. Kojima |
| Toho University Sakura Medical Center | N. Kamiya |
| Keio University Hospital | R. Mizuno |
| Tokyo Medical and Dental University | Y. Fujii |
| Nippon Medical School Hospital | G. Kimura |
| Kitasato University School of Medicine | T. Fujita |
| Yokohama City University Hospital | N. Nakaigawa |
| Niigata University Medical and Dental Hospital | Y. Tomita |
| University of Toyama, University Hospital | Y. Ikehata |
| Shizuoka Cancer Center | R. Yamashita |
| Hamamatsu University School of Medicine | H. Miyake |
| Aichi University Medical Hospital | T. Tsuzuki |
| Nagoya University Hospital | N. Sassa |
| Kyoto University Graduate School of Medicine | O. Ogawa |
| Osaka City University Hospital | S. Tamada |
| Osaka University Hospital | M. Uemura |
| Kindai University Hospital | M. Nozawa |
| Kobe University Hospital | K. Harada |
| Nara Medical University Hospital | S. Anai |
| Okayama University Hospital | A. Takamoto |
| Tokushima University Hospital | T. Fukawa |
| Kagawa University | M. Sugimoto |
| Kyushu University Hospital | K. Tatsugami |
| Nagasaki University Hospital | H. Sakai |
| Kumamoto University Hospital | T. Motoshima |
